# Genomic dissection of plant development and its impact on thousand grain weight in barley through nested association mapping

**DOI:** 10.1093/jxb/erw070

**Published:** 2016-03-01

**Authors:** Andreas Maurer, Vera Draba, Klaus Pillen

**Affiliations:** ^1^Institute of Agricultural and Nutritional Sciences, Martin Luther University Halle-Wittenberg, Betty-Heimann-Str. 3, 06120 Halle, Germany.; ^2^Interdisciplinary Center for Crop Plant Research (IZN), Betty-Heimann-Str. 3, 06120 Halle, Germany.

**Keywords:** Barley, flowering time, genome-wide association study (GWAS), nested association mapping (NAM), plant development, quantitative trait locus (QTL), thousand grain weight, wild barley.

## Abstract

Flowering time is a key agronomic trait that plays an important role in crop yield. There is growing interest in dissecting the developmental subphases of flowering to better understand and fine-tune plant development and maximize yield. To do this, we used the wild barley nested association mapping (NAM) population HEB-25, comprising 1420 BC_1_S_3_ lines, to map quantitative trait loci (QTLs) controlling five developmental traits, plant height, and thousand grain weight. Genome-wide association studies (GWAS) enabled us to locate a total of 89 QTLs that genetically regulate the seven investigated traits. Several exotic QTL alleles proved to be highly effective and potentially useful in barley breeding. For instance, thousand grain weight was increased by 4.5g and flowering time was reduced by 9.3 days by substituting Barke elite QTL alleles for exotic QTL alleles at the *denso/sdw1* and the *Ppd-H1* loci, respectively. We showed that the exotic allele at the semi-dwarf locus *denso/sdw1* can be used to increase grain weight since it uncouples the negative correlation between shoot elongation and the ripening phase. Our study demonstrates that nested association mapping of HEB-25 can help unravel the genetic regulation of plant development and yield formation in barley. Moreover, since we detected numerous useful exotic QTL alleles in HEB-25, we conclude that the introgression of these wild barley alleles into the elite barley gene pool may enable developmental phases to be specifically fine-tuned in order to maximize thousand grain weight and, potentially, yield in the long term.

## Highlight

The genetic control of plant development was investigated in a multi-parental wild barley NAM population. We found that major flowering genes control plant development and highlight trait-improving exotic alleles.

## Introduction

In the past few decades the phenotypic characterization and genetic dissection of flowering time has been achieved for numerous model species and crops (Blümel *et al.*, 2015). Barley (*Hordeum vulgare* ssp. *vulgare* L.) has been established as a model species for temperate cereals and serves as a solid base for dissecting the genetic basis of flowering time regulation. In this regard, day length (photoperiod) and sensitivity to cold temperatures (vernalization) have been identified as being two major determinants of flowering time. *Vrn-H3* (Yan *et al.*, 2006) is the key gene controlling flower initiation in barley. It is an orthologue of the Arabidopsis *FT* (*FLOWERING LOCUS T*) gene. The FT protein moves from leaves to the shoot apical meristem, as postulated decades ago for the mobile signal ‘florigen’ (Chailakhyan, 1937). Its function as a promoter of flowering is assumed to be preserved across different plant species (Turck *et al.*, 2008).

The key vernalization genes *Vrn-H1* (Yan *et al.*, 2003) and *Vrn-H2* (Yan *et al.*, 2004) have a major impact on *Vrn-H3* expression. *Vrn-H2* was determined to be a repressor of *Vrn-H3*, which prevents flowering under long days before vernalization. *Vrn-H1* responds to low temperatures (Oliver *et al.*, 2013) as a result of *cis*-regulatory elements in its promotor region (Alonso-Peral *et al.*, 2011). It is up-regulated after vernalization and, thus, promotes flowering through direct binding to the promoters of *Vrn-H2* (repression) and *Vrn-H3* (activation) (Deng *et al.*, 2015). A differentiation between winter and spring barley can be made based on their response to vernalization. The latter lacks the vernalization requirement as a result of a natural deletion of *Vrn-H2* (von Zitzewitz *et al.*, 2005).

Flowering is furthermore promoted by *Ppd-H1* under long days (Turner *et al.*, 2005) and by *Ppd-H2* under short days (Kikuchi *et al.*, 2009). In addition to photoperiod and vernalization, light quality (Nishida *et al.*, 2013; Pankin *et al.*, 2014), circadian rhythms (Campoli *et al.*, 2012; Faure *et al.*, 2012; Zakhrabekova *et al.*, 2012; Campoli *et al.*, 2013; Calixto *et al.*, 2015), and phytohormones like gibberellic acid (GA) (Jia *et al.*, 2009; Jia *et al.*, 2011; Boden *et al.*, 2014) and cytokinins (Mrízová *et al.*, 2013) also contribute to the induction of flowering in barley. Many loci that were previously classified as earliness *per se* genes that act independently from external signals (Laurie *et al.*, 1995) were recently assigned to those classes. The complexity of floral networks (Blümel *et al.*, 2015) is caused by the interplay between numerous genes and external signals. It is worth taking a closer look at specific developmental subphases to help unravel these networks.

The life cycle of barley consists of several subphases. The most basic differentiation divides the life of a barley plant into vegetative, reproductive and grain-filling phases (Sreenivasulu and Schnurbusch, 2012). Vegetative plant organs develop during the vegetative phase. The reproductive phase starts with the initiation of spikelets, which develop over time. This phase is terminated with anthesis resulting in the onset of the grain-filling phase. The length of different preanthesis subphases has been shown to be under genetic control (Kernich *et al.*, 1997; Borras *et al.*, 2009; Borras-Gelonch *et al.*, 2010; Borras-Gelonch *et al.*, 2012; Alqudah *et al.*, 2014) and impacts yield-related traits (Miralles *et al.*, 2000; Alqudah and Schnurbusch, 2014, 2015). The postanthesis phase is also assumed to have a major impact on yield by determining the time frame for grain-filling (Evans and Wardlaw, 1976; Egli, 2004), which controls the yield component grain weight (Distelfeld *et al.*, 2014). Although the timing and duration of developmental phases is important for a plant’s yield potential, most genomic studies dealing with the regulation of plant development and its impact on yield focus solely on flowering time as the only developmental parameter.

Nested association mapping (NAM) has been shown to be a valuable tool in the dissection of the genetic architecture of many traits in maize, sorghum, and barley (Buckler *et al.*, 2009; Jordan *et al.*, 2011; Maurer *et al.*, 2015). A NAM population is the result of wide crosses of highly diverse donor genotypes with a recurrent elite cultivar, followed by several rounds of selfing. It combines the advantages of association mapping (i.e. high allele richness and mapping resolution) and linkage mapping (i.e. high statistical power). It also provides excellent opportunities to evaluate the performance of untapped wild alleles in an elite background as a result of its exclusive mating design (Ogut *et al.*, 2015).

The aim of this study was to characterize the genetic architecture of barley development. To do this, we used the NAM population HEB-25 (Maurer *et al.*, 2015) consisting of 1420 highly divergent BC_1_S_3_ lines. We also wanted to shed more light on the flowering time pathway in barley by comparing the impact flowering time genes had on different developmental subphases. In addition to time to flowering (HEA), we investigated time to shooting (SHO), duration of the shoot elongation phase (SEL), duration of the ripening phase (RIP) and time to maturity (MAT). In order to gain insight into additional physiological functions of flowering time genes and their impact on yield formation, we also investigated plant height (HEI) and thousand grain weight (TGW, Table 1). Furthermore, we looked at whether there was useful variation present in the wild barley germplasm that could be used to fine-tune specific developmental phases in order to increase yield potential in future barley breeding programmes.

**Table 1.** List of evaluated traits

Abbreviation	Trait	Unit	Method of measurement	Years studied
SHO	Shooting	days	Number of days from sowing until first node palpable at least 1cm above the tillering node for 50% of all plants of a plot (BBCH 31; Lancashire *et al.*, 1991)	2011–2014
SEL	Shoot elongation phase	GDD	Time from SHO to HEA	2011–2014
HEA	Flowering	days	Number of days from sowing until first awns visible (BBCH 49; Lancashire *et al.*, 1991) for 50% of all plants of a plot	2011–2014
RIP	Ripening phase	days	Time from HEA to MAT	2012–2014
MAT	Maturity	days	Number of days from sowing until hard dough: grain content solid and fingernail impression held (BBCH 87; Lancashire *et al.*, 1991) for 50% of all plants of a plot	2012–2014
TGW	Thousand grain weight	g	Calculated after harvest by use of MARVIN seed analyser (GTA Sensorik GmbH, Neubrandenburg, Germany) based on a 200 seeds sample of each plot. Before, seeds were cleaned and damaged seeds were sorted out	2011–2013
HEI	Plant height	cm	Recorded at maturity as the distance from ground to tip of the erected ear (without awns), taken as an average across the ears of a plot	2011–2013

## Materials and methods

### Plant material

The NAM population HEB-25 (Maurer *et al.*, 2015), consisting of 1420 individual BC_1_S_3_ lines in 25 wild-barley-derived subfamilies, was used in this study. HEB-25 was the result of initial crosses between the spring barley cultivar Barke (*Hordeum vulgare* ssp. *vulgare*) and 25 highly divergent exotic wild barley accessions (*Hordeum vulgare* ssp. *spontaneum* and *agriocrithon*). F_1_ plants of the initial crosses were backcrossed with Barke. For detailed information about the population design, see Maurer *et al.* (2015).

### Collecting single nucleotide polymorphism data

Single nucleotide polymorphism (SNP) genotype data were collected at TraitGenetics, in Gatersleben, Germany, for all 1420 individual BC_1_S_3_ lines and their corresponding parents. The barley Infinium iSelect 9K chip consisted of 7864 SNPs (Comadran *et al.*, 2012). The genotype data were processed and stored as indicated in Maurer *et al.* (2015) and 5709 informative SNPs, which met the quality criteria, could be utilized in this study. An identity-by-state approach was used to differentiate between the HEB genotypes. Based on parental genotype information, the exotic allele could be specified in each segregating family, and homozygous exotic genotypes were assigned a value of 2. HEB lines that showed a homozygous Barke genotype were assigned a value of 0. Consequently, heterozygous HEB lines were assigned a value of 1. Numbers can therefore be interpreted as a quantitative variable representing the dose of the wild allele.

### HEB-25 field trials

Between 2011 and 2014, four field trials were conducted at the ‘Kühnfeld Experimental Station’ of Martin Luther University Halle-Wittenberg (51°29′46.47″N; 11°59′41.81″E) to gather phenotype data. In 2011, the field trial was conducted with selfed progenies of BC_1_S_3_ lines (so-called BC_1_S_3:4_), arranged as a single randomized block. The majority of plots (92%) included one or two rows per HEB line whereas the remaining 8% of plots included three to five rows, depending on the number of available BC_1_S_3:4_ seeds. Plots in 2011 had a length of 1.50 m, a distance of 0.20 m between rows, and were separated by 0.50 m to reduce competition between plots. In 2012 and 2013, the field trials were conducted with the selfed progenies in BC_1_S_3:5_ and BC_1_S_3:6_, respectively. Two replications per HEB line, arranged in two randomized complete blocks, were cultivated in 2012 and 2013. The plots consisted of two rows (30 seeds each) with a length of 1.50 m, a distance of 0.20 m between rows, and a spacing of 0.50 m between plots. In 2014, BC_1_S_3:7_ seeds were sown in a single randomized block. The plots consisted of two rows (50 seeds each) with a length of 1.50 m, a distance of 0.20 m between rows and a spacing of 0.50 m between plots. Barke was integrated as a check line in all trials. All field trials were sown in spring, between March and April, with fertilization and pest management carried out according to local practice. No additional fertilizer was applied in 2014.

### Phenotypic data

Table 1 shows a list of all of the investigated traits and a description of their measurements and the years studied. This information is supplemented insofar as all developmental traits were recorded both as ‘days from sowing’ and ‘growing degree days’ (GDD), which were calculated with a base temperature of 0 °C in accordance with equation (1) in McMaster and Wilhelm (1997). Thus, the mean daily temperatures of all of the days with a mean temperature above 0 °C were cumulated.

### Statistical analyses

We performed a one-step phenotypic data analysis for all traits with SAS 9.4 (SAS Institute Inc., Cary, NC, USA), based on a linear mixed model (PROC MIXED) with effects for genotype (i.e. 1420 HEB lines), environment (i.e. 4 years) and interaction of genotype and environment. To estimate variance components, all effects were assumed to be random (PROC VARCOMP). Heritabilities across years were calculated as: $h^{2}=\frac{V_{\text{G}}}{V_{\text{G}}\;+\;\frac{V_{\text{GY}}}{y}\;+\;\frac{V_{\text{R}}}{yr}}$ ,

where *V*_G_, *V*_GY_ and *V*_R_ represent the genotypic components, genotype × year, and error variance components, respectively. The terms *y* and *r* indicate the number of years and replicates, respectively. Best linear unbiased estimates (BLUEs) were calculated with PROC MIXED for each genotype assuming fixed genotype effects. BLUEs were used to calculate Pearson’s correlation coefficients (*r*) with PROC CORR.

### Genome-wide association study

We applied Model B on trait BLUEs as outlined in detail by Liu *et al.* (2011). This model was found to be most suited for carrying out genome-wide association study (GWAS) with multiple families (Würschum *et al.*, 2012) and has already been shown to work properly in HEB-25 (Maurer *et al.*, 2015). It is based on multiple regression, taking into account a quantitative SNP effect and a qualitative family effect in addition to quantitative cofactors that control both population structure and genetic background (Würschum *et al.*, 2012). Cofactor selection was carried out on this model and included all SNPs simultaneously by applying PROC GLMSELECT in SAS and minimizing the Schwarz Bayesian Criterion (Schwarz, 1978). PROC GLM was used to perform the genome-wide scan for the presence of marker–trait associations. Cofactors that were linked closer than 1 cM to the SNP under investigation were excluded. The Bonferroni–Holm method (Holm, 1979) was used to adjust marker–trait associations for multiple testing. Significant marker effects were accepted with *P*_BON-HOLM_<0.05. The proportion of the phenotypic variance explained by a marker was determined by estimating *R* ^2^ after modelling the marker solely in a linear model. Additive effects for each SNP were taken as the regression coefficient of the SNP directly from the GWAS model. Family-specific effects were calculated for all markers based on a simple linear model, including a general family term and the marker effect as nested within the family.

To increase the robustness of the method, the entire procedure was applied 200 times on random subsamples of the full dataset. Each subsample included 80% of the lines, randomly selected per HEB family. We recorded the significant (*P*_BON-HOLM_<0.05) markers detected, which is referred to as the detection rate. Markers that were detected in at least 10% of subsamples were accepted as putative QTLs, following Ogut *et al.* (2015). Significant markers were merged into a single QTL if they were linked by less than 4 cM. Additive effects, *P*_BON-HOLM_ values and *R* ^2^ were averaged across all runs, in which the respective marker was significant. In order to evaluate the explained phenotypic variance, the unbiased estimator *R* ^2^_adj_ (Draper and Smith, 1981) was determined for each subsample by simultaneously modelling all of the significant markers in a linear model. In order to determine the predictive ability *R* ^2^_pred_, the estimated additive effects of each subsample were used to predict the phenotypic value of the remaining 20% of the lines. We then calculated *R* ^2^_pred_ to be the squared Pearson product–moment correlation between predicted and observed phenotypic values. The means of *R* ^2^_adj_ and *R* ^2^_pred_, measured over 200 runs, were ultimately recorded as the final values.

We used the BARLEYMAP pipeline (Cantalapiedra *et al.*, 2015) to identify potential candidate genes to explain the QTLs. BARLEYMAP enables markers to be mapped and gene sequences to be aligned against sequence-enriched genetic (Mascher *et al.*, 2013) and physical frameworks (International Barley Genome Sequencing Consortium, 2012). This represents a very precise way to find positional coincidence of QTLs with putative candidate genes. The results are presented in Supplementary Table S1 at *JXB* online. We also compared our wild allele effects to those reported in a barley BC_2_DH population and a set of wild barley introgression lines (Wang *et al.*, 2010). Here several known flowering time genes were sequenced in wild and cultivated barley. Since the wild allele effect was also produced directly from a cross of wild barley and cultivated barley, we assume that similar effects indicate the same candidate genes.

## Results and discussion

### Phenotypes

A broad variation in phenotypes of HEB-25 lines was observed for all traits across and within years. This resulted in high coefficients of variation (Supplementary Table S2). After calculating BLUEs, which were corrected for year effects, we observed large differences of more than 100% between the most extreme HEB lines for each trait except MAT (Table 2 and Supplementary Fig. S1). For instance, a difference of 51.4 days between the earliest and the latest genotypes could be observed for HEA, and likewise TGW varied between 19.4g and 60.2g. The lowest coefficient of variation was obtained for the trait MAT, where the most extreme genotypes nevertheless showed a difference of 33.4 days.

**Table 2.** Descriptive statistics for best linear unbiased estimates (BLUEs) and heritabilities across all environments

Trait^*a*^	N^*b*^	Mean^*c*^	SD^*d*^	Min^*e*^	Max^*f*^	CV%^*g*^	*h* ^2 *h*^
SHO	1422	53.3	5.6	38.6	82.6	10.4	0.93
SEL	1422	237.8	42.6	108.9	396.0	17.9	0.75
HEA	1422	67.9	6.3	50.4	101.9	9.2	0.94
RIP	1420	32.7	2.6	19.2	40.5	7.9	0.81
MAT	1420	101.3	4.5	88.5	121.9	4.5	0.91
TGW	1422	46.5	5.0	19.4	60.2	10.8	0.57
HEI	1420	63.9	8.5	41.0	100.5	13.3	0.88

^*a*^ Trait abbreviations are given in Table 1.

^*b*^ Number of observations (genotypes).

^*c*^ Arithmetic mean.

^*d*^ Standard deviation.

^*e*^ Minimum.

^*f*^ Maximum.

^*g*^ Coefficient of variation (%).

^*h*^ Heritability.

### Phenotypic correlations

We calculated Pearson’s correlation coefficients (*r*) in order to gain basic insights into the relationships between the different phases of plant development, HEI, and their influence on TGW. We observed very high correlations (0.88–0.93) between SHO, HEA, and MAT (Table 3). This indicates that early shooting lines also tend to be early for other stages. Another peculiarity is that in the case of RIP correlation coefficients were negative for all other developmental stages. Together with the relatively low coefficient of variation for MAT, this indicates that the time of maturity may be predetermined or limited to some extent by environmental factors such as heat and drought. As both SEL and RIP were calculated to be the difference between two other stages, we can draw conclusions about their main determinant. Following this, HEA had the greatest impact on the duration of SEL and RIP, since its correlation coefficients outperformed those of SHO and MAT, respectively. The duration of SEL greatly impacted HEI (*r*=0.45), indicating that the occurrence of (semi-)dwarf plants was based more on a shortened period of SEL than on a reduced growth rate. No correlation with TGW could be observed for SEL. TGW was positively correlated with RIP (*r*=0.37), which may be due to an extension of grain-filling, where starch is being stored in the grains (Distelfeld *et al.*, 2014). Interestingly, HEI was also positively correlated with TGW (*r*=0.31).

**Table 3.** *Pearson’s correlation coefficients* (r)

	SEL	HEA	RIP	MAT	TGW	HEI
SHO	**0.32**	**0.92**	**–0.67**	**0.88**	**–0.38**	–0.01
SEL		**0.66**	**–0.60**	**0.57**	–0.07	**0.45**
HEA			**–0.79**	**0.93**	**–0.35**	**0.17**
RIP				**–0.54**	**0.37**	**–0.19**
MAT					**–0.28**	**0.13**
TGW						**0.31**

Bold values indicate significant correlations at *P*<0.0001. Trait abbreviations are given in Table 1.

### Heritabilities

Heritabilities for all investigated traits were calculated over 3–4 years. We observed heritabilities >0.5 for all traits (Table 2). Heritabilities for the developmental traits SHO, HEA, and MAT were almost identical, regardless of whether they were measured in days or growing degree days (GDD) (Supplementary Table S3). However, days outperformed GDD (0.81 vs 0.68) in the case of RIP, while GDD outperformed days (0.75 vs 0.69) in the case of SEL. Thus, for SEL, GDD rather than days offers a better estimate for the relative time needed to fulfil this stage, since plant growth rate and, hence, plant development is based on the interplay between different temperature-dependent biochemical processes (Atwell *et al.*, 1999; Atkin and Tjoelker, 2003). This is of particular importance, especially when dealing with data from different years and when taking into account that the onset of SEL differs greatly (from 38.6 to 82.6 days after sowing) between individual lines in the HEB-25 population. In contrast, the use of days instead of GDD resulted in a higher heritability for RIP. This observation may be attributed to the fact that plant development does not benefit from higher temperatures if a certain temperature threshold is met or the plant has reached a critical physiological state. McMaster and Smika (1988) compared temperature-dependent and -independent models to predict growth stages in winter wheat and pointed out that the best model for predicting a developmental stage varied depending on the respective stage. Therefore, we decided to concentrate on GDD for SEL and days for all other developmental traits in our analyses.

### GWAS

We conducted GWAS for each trait in order to further analyse the above-mentioned correlations between traits and to elucidate which QTLs are responsible for controling trait variation in HEB-25. We applied a multiple linear regression model, including a population main effect and selected markers as cofactors, to account for genetic background and relatedness. This model was recently shown to perform best in HEB-25 (Maurer *et al.*, 2015).

**Fig. 1.**
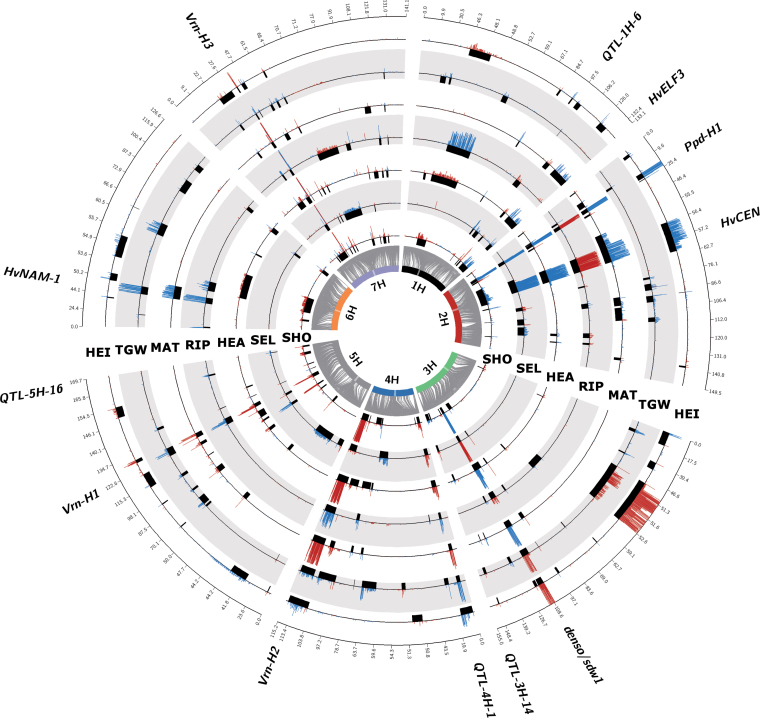
**Comparison of GWAS results across developmental traits, thousand grain weight and plant height.** Barley chromosomes are indicated as coloured bars on the inner circle, and centromeres are highlighted as transparent boxes. Grey connector lines represent the genetic position of SNPs on the chromosomes, which is given in centimorgans on the outer circle. Each track represents one trait, and these are (from inside to outside) SHO, SEL, HEA, RIP, MAT, TGW and HEI. Trait abbreviations are given in Table 1. Black boxes indicate the QTL positions. The height of histogram bars above represent the detection rate across 200 repeated random subsamples. The blue and red colours of the bars indicate trait-reducing and trait-increasing effects, respectively, exerted by exotic QTL alleles. Candidate genes of major QTLs are indicated outside the circle.

We were able to detect numerous associated genomic regions for all of the traits studied using our GWAS method (Fig. 1 and Supplementary Table S4). A total of 89 QTLs could be defined (Table 4 and Supplementary Table S5). Most QTLs were shared by multiple traits. However, we could also detect trait-specific QTLs for all of the seven traits (Supplementary Fig. S2). We obtained broad variation for wild allele QTLs, with an increase or decrease in trait values compared with the Barke control allele. In this regard, we were able to identify 10 QTLs where the wild allele increased TGW by up to 6.6g (Supplementary Table S5). Modelling all significant markers of one trait simultaneously resulted in explained percentages of phenotypic variance (*R* ^2^_adj_) ranging from 63.6% (TGW) to 82.3% (HEI). The fact that *R* ^2^_pred_ values, resulting from a prediction of phenotypes of an independent sample, were also comparatively high (Table 4) confirms the robustness of the method and indicates that a large fraction of phenotypic variation can be explained by genotypic variation.

**Table 4.** Number of QTLs and total explained phenotypic variance

Trait^*a*^	QTLs (*n*)^*b*^	*R* ^2^_adj_ (%)^*c*^	*R* ^2^_pred_ (%)^*d*^
SHO	49	81.6	59.9
SEL	28	65.9	42.5
HEA	43	79.0	55.5
RIP	30	66.6	37.5
MAT	32	76.9	56.2
TGW	43	63.6	42.8
HEI	37	82.3	45.7
No. of unique QTLs	89		

^*a*^ Trait abbreviations are given in Table 1.

^*b*^ Number of QTLs detected for the respective trait.

^*c*^ Mean explained phenotypic variance by GWAS.

^*d*^ Mean ability to predict phenotypes of an independent sample.

**Fig. 2.**
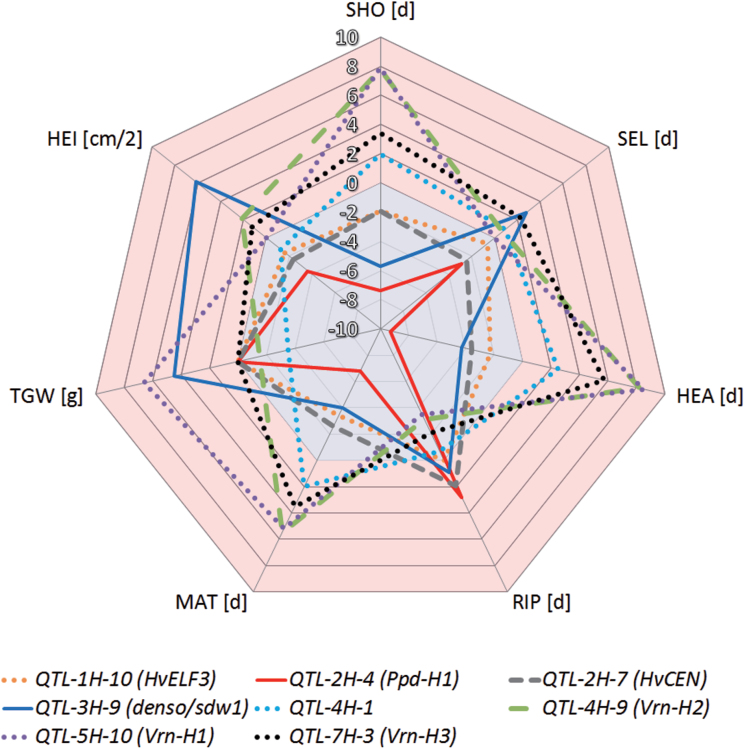
**Spider diagram of major QTL effects across traits.** Different traits are represented by corner points of the net. Trait abbreviations are given in Table 1. Effects of wild alleles are indicated by differentially shaped lines for the respective QTL. The blue-shaded and red-shaded backgrounds of the spider net indicate trait-reducing and trait-increasing effects, respectively, exerted by exotic QTL alleles. To enable a comparison of traits within the same scale, values of SEL have been divided by 16.3, which represents the equivalent of one GDD to one day during SEL. Values of HEI have been divided by 2.

Below, we present eight common QTLs (starting with chromosome 1H in ascending order) and discuss their relevance in controlling the five developmental traits SHO, SEL, HEA, RIP, and MAT. Then, in order to draw conclusions about their potential QTL function, we study their effects on HEI and TGW. Since HEB-25 enables high genetic precision in mapping of QTLs (Maurer *et al.*, 2015), we obtained strong positional coincidence with plausible candidate genes (Supplementary Table S1). We therefore directly refer to these candidate genes in the headings of the subsections. The results of these eight major QTLs are summarized in Table 5 and illustrated in Fig. 2. Finally, we discuss the developmental phase-specific QTLs that were obtained in this study.

**Table 5.** Major developmental QTLs and their impact on further traits

			cM interval^*c*^		SHO^*d*^	SEL	HEA	RIP	MAT	TGW	HEI	Candidate gene/locus with reference	
QTL	Chr^*a*^	Peak marker^*b*^	From	Until									
QTL-1H-10	1H	i_SCRI_RS_150786	128.0	133.1	**–1.9**	**–10.2**	**–2.3**		**–3.4**		**–3.2**	*HvELF3*	( Faure *et al.*, 2012; Zakhrabekova *et al.*, 2012)
QTL-2H-4	2H	i_BK_12	22.2	23.8	**-7.4**	**–46.7**	**–9.3**	2.8	**–6.8**		**–7.3**	*Ppd-H1*	( Turner *et al.*, 2005)
QTL-2H-7	2H	i_12_30265	53.3	60.8	**–1.9**	**–39.8**	**–3.6**	1.9	**–2.6**		**–4.7**	*HvCEN*	( Comadran *et al.*, 2012)
QTL-3H-9	3H	i_11_11172	103.8	109.8	**–5.7**	44.9	**–4.3**	0.9	**–4.0**	4.5	12.3	*denso/sdw1*	( Jia *et al.*, 2009)
QTL-4H-1	4H	i_12_31458	0.6	14.9	2.0	15.4	2.5	**–0.7**	2.0	**–3.5**	**–2.6**		
QTL-4H-9	4H	i_SCRI_RS_216897	110.2	114.3	7.8	11.3	8.2	**–3.1**	5.5	**–1.5**	4.2	*Vrn-H2*	( Yan *et al.*, 2004)
QTL-5H-10	5H	i_11_10783	122.4	128.5	7.9		8.5	**–3.5**	5.2	6.6		*Vrn-H1*	( Yan *et al.*, 2003)
QTL-7H-3	7H	i_12_30895	29.8	34.3	3.4	36.8	5.7	**–2.1**	3.5		2.5	*Vrn-H3*	( Yan *et al.*, 2006)

Negative values are indicated in bold. Blank cells indicate that the respective QTL was not detected for the trait. Trait abbreviations are given in Table 1. For a complete table of all QTLs, see Table S5.

^*a*^ Chromosome on which the QTL was detected.

^*b*^ iSelect name of marker with the highest significance for HEA.

^*c*^ Genetic interval (cM) with lower and upper threshold of QTL, based on the map of Maurer *et al.* (2015).

^*d*^ Most extreme effect (absolute difference of homozygous wild genotype and homozygous cultivated genotype) of all significant SNPs in respective QTL interval.

### QTL-1H-10 (*HvELF3*)

We observed a QTL that showed significant effects for all seven traits studied except for RIP and TGW. The QTL is located close to the telomere of chromosome arm 1HL (QTL-1H-10). The exotic allele at this locus is associated with a slight acceleration of plant development. The time to reach each stage was shortened by 2–3 days in contrast to the allele of the spring barley cultivar Barke. This QTL region harbours the earliness-inducing *eam8/mat-a* locus (Franckowiak *et al.*, 1997) and may correspond to *HvELF3* (*EARLY FLOWERING3*), which is orthologous to the Arabidopsis circadian clock gene *ELF3* (Faure *et al.*, 2012; Zakhrabekova *et al.*, 2012). *HvELF3* was recently shown to influence flowering by regulating GA production in barley (Boden *et al.*, 2014).

### QTL-2H-4 (*Ppd-H1*)

QTL-2H-4 exerted significant effects on all developmental traits and HEI, thereby explaining up to 34% of the phenotypic variance (*R* ^2^). The most significant SNP marker (i_BK_12) is directly located within the *Ppd-H1* gene, which is the main determinant of response to long day conditions in barley (Turner *et al.*, 2005). Most wild barley accessions were shown to carry a highly responsive *Ppd-H1* allele, accelerating development under long day conditions (Cockram *et al.*, 2011). In contrast, Barke, like most spring barley from Northern Europe, carries an allele that exhibits a reduced response to long days. In our study, the wild allele led to a strong acceleration of all developmental phases by up to 9.3 days, except for RIP, which was delayed by up to 3 days. In contrast, HEI was reduced by 7.3cm when the wild *Ppd-H1* allele was present. After reaching the reproductive stage, relatively more energy is put into reproductive organs. This leads to reduced vegetative growth. Thus, the wild allele at *Ppd-H1* may accelerate floral development at the expense of growth and biomass production.

### QTL-2H-7 (*HvCEN*)

We observed a QTL, located next to the centromere of chromosome 2H, that showed significant effects on all traits except TGW. Comadran *et al.* (2012) identified *HvCEN* (*CENTRORADIALIS*), a homologue of Arabidopsis *TFL1* (*TERMINAL FLOWER1*), as the possible gene behind this locus. Recently, Loscos *et al.* (2014) found evidence that *HvCEN* plays a central role in the induction of flowering in barley. The effects of *HvCEN* on all developmental phases were similar to the effects of *Ppd-H1*. However, the scale of the effects was clearly reduced. At this point we should note that the *HvCEN* effect is presumably not due to genetic linkage to *Ppd-H1*, since *Ppd-H1* was selected as a cofactor in the GWAS model for calculating the *HvCEN* association.

### QTL-3H-9 (*denso/sdw1*)

QTL-3H-9 was detected for all five developmental traits. This QTL may correspond to the *denso/sdw1* locus, causing a semi-dwarf phenotype. *HvGA20ox2*, coding a GA-20 oxidase enzyme, is a candidate gene for explaining its function (Jia *et al.*, 2009; Jia *et al.*, 2011). The *denso* allele is commonly present in modern European malting barley cultivars like Barke (Jia *et al.*, 2009). Therefore, it is not surprising that we also observe a strong effect on HEI for this locus, explaining 41% of phenotypic variance (*R* ^2^). The presence of the wild allele increased HEI by up to 12.3cm. This QTL simultaneously affected all developmental traits and TGW. The wild allele reduced the time required to reach SHO, HEA and MAT by 5.7, 4.3 and 4.0 days, respectively. At the same time it delayed the time between these stages (SEL and RIP). Furthermore, the wild allele increased TGW by up to 4.5g. As the absolute effects steadily diminished throughout the developmental stages (Table 5), we presume that *denso/sdw1* plays a major role in the very early stages of development. All in all, the influence of *denso/sdw1* on all investigated traits underlines its complex and important role in plant physiology throughout a plant’s life cycle—a known feature of GA (Taiz and Zeiger, 2007). In addition to the importance of GA in floral regulatory networks (Mutasa-Göttgens and Hedden, 2009), elevated levels of GA also play a role in the delay of senescence, as shown for pea (Wang *et al.*, 2007). Furthermore, GA levels were shown to be crucial for endosperm differentiation and for the growth of barley grains (Weier *et al.*, 2014).

### QTL-4H-1

QTL-4H-1 is another ubiquitous QTL that has recently been identified as being a major QTL for flowering time in barley (Maurer *et al.*, 2015). The position of the most significant peak markers differs between 3.5 and 14.9 cM depending on the investigated trait. Nevertheless we assume that both peaks correspond to the same QTL due to a lack of marker density in this region. This QTL has also been found for TGW and HEI, underscoring its crucial role in plant physiology. This QTL delayed all developmental stages by approximately 2 days, except for RIP, which was accelerated by approximately 2 days. Simultaneously, TGW was reduced by 3.5g and HEI was reduced by 2.6cm. To date, no clear candidate gene for this QTL has been found. However, after aligning the sequences of significant markers in this region against sequence-enriched genetic and physical frameworks via BARLEYMAP (Cantalapiedra *et al.*, 2015) a *CCT* (*CONSTANS*, *CO*-like, and *TOC1*) domain gene was identified in this region (Genbank accession number AK354746). Many known flowering time regulators have *CCT* domains (Cockram *et al.*, 2012). Furthermore, the *LOG* (*LONELY GUY*) gene (MLOC_45038.2) could be assigned to this region. *LOG* encodes a cytokinin-activating enzyme that is required to maintain meristem activity. Its loss of function causes pre-mature termination of the shoot meristem in rice development (Kurakawa *et al.*, 2007).

### QTL-4H-9 (*Vrn-H2*)

QTL-4H-9, which may harbour *Vrn-H2*, the main repressor of flowering in vernalization-dependent barley (Yan *et al.*, 2004), was reliably detected for SHO, HEA, RIP and MAT. The wild allele delayed the respective developmental stages by up to 8 days, whereby RIP was shortened by up to 3 days. However, the high variation of marker effects among significant SNPs indicates that effects vary greatly depending on the segregating families of the respective SNPs (Supplementary Table S4). This locus is naturally deleted in spring barley cultivars like Barke. The result is that no vernalization is required to induce flowering (von Zitzewitz *et al.*, 2005). This explains why the alleles from wild barley, which are predominantly winter types (Cockram *et al.*, 2011), delayed flowering in our spring-sown field trials. The fact that this QTL was infrequently detected for the traits SEL, TGW and HEI leads us to assume that the impact of *Vrn-H2* on these traits may be diminished or biased by family-specific effects.

### QTL-5H-10 (*Vrn-H1*)

This QTL was shared between SHO (+7.9 d), HEA (+8.5 d), RIP (–3.5 d) and MAT (+5.2 d) and may correspond to the *Vrn-H1* locus with *HvBM5A* (*MADS-box 5A*) being a candidate gene (von Zitzewitz *et al.*, 2005). *Vrn-H1* is known to be involved in the vernalization pathway of flowering time regulation by responding to the low temperatures required for vernalization (Oliver *et al.*, 2013). *HvPhyC* (*PHYTOCHROME C*) is another gene that affects flowering time (Nishida *et al.*, 2013; Pankin *et al.*, 2014). It is closely linked to *HvBM5A*, which makes it difficult to distinguish between their effects. In addition, a QTL for HEI (QTL-5H-11) has been defined approximately 3 cM distal from the position of *HvBM5A* and *HvPhyC.* This further complicates the interpretation of the QTL effects in this genomic region. Again, as already shown for *Vrn-H2*, the high variation of marker effects points to family-specific effects for this locus. An extraordinarily high variation exists for the trait TGW, which is caused by a single SNP marker (i_11_11090) that is only polymorphic in HEB-F08. The wild allele caused an estimated increase in TGW by 6.6g, whereas all other significant markers in the *Vrn-H1* region reduced TGW by 1.5–1.8g. Interestingly, there is low variation for the locus affecting HEI, located 3 cM distal from *Vrn-H1.* This indicates that this locus might be independent of *Vrn-H1*.

### QTL-7H-3 (*Vrn-H3*)

The most significant SNP marker at this QTL (i_12_30895) is directly located within *Vrn-H3*. The *Vrn-H3* locus in barley has been shown to correspond to *HvFT1*, the orthologue of Arabidopsis *FT* (Yan *et al.*, 2006; Faure *et al.*, 2007). This gene plays a central role in the flowering pathways as it is involved in the switch from vegetative to reproductive growth under long day conditions (Turck *et al.*, 2008). In our population, significant effects of this QTL were observed for all developmental traits and HEI. On average, the wild allele in the *Vrn-H3* region delayed all developmental phases by 3–6 days, except RIP, which was shortened by approximately 2 days. However, ample variation in marker effects exists for all developmental traits (Supplementary Table S4). For instance we observed effects for SHO ranging from –3.3 to +3.4 days depending on the families in which the respective marker segregates. This clearly shows the presence of family-specific effects, most likely due to functionally different haplotypes of *Vrn-H3* among the HEB-25 donor accessions. However, for the trait HEI (increased by 2.5cm) the effect of the wild allele was relatively constant. Interestingly, gene-specific markers of *Vrn-H3* did not impact TGW. Instead, the QTL for TGW was defined at around 4 cM proximal of *Vrn-H3* at 38.8 cM (QTL-7H-4) causing a reduction of TGW by up to 2.2g.

### Developmental phase-specific QTLs

In addition to the majority of QTLs that were shared across developmental traits, we also found certain QTLs that impacted a specific stage of plant development. These QTLs are a potential source for fine-tuning the phases of plant development and unravelling the physiological pathways.

### QTLs affecting early development

QTLs that affect early development are characterized by their influence on SHO and SEL. SEL is strongly related to the phase from awn primordium to tipping, which was shown to be the most decisive developmental phase for spikelet survival (Alqudah and Schnurbusch, 2014) and leaf growth rate (Alqudah and Schnurbusch, 2015) in barley. The length of this phase is therefore assumed to play a key role in determining a plant’s yield potential.

We found three striking QTLs (QTL-1H-6, QTL-3H-14 and QTL-5H-16) that exhibited exclusive effects on SHO and SEL. In the case of QTL-3H-14, SHO was accelerated by approximately 2 days and TGW increased by 1.3g. In the case of QTL-1H-6 and QTL-5H-16, SHO was delayed by 1–2 days without any effect on TGW. These QTLs were not shown to affect HEA since the effect on SEL was contrary to the effect on SHO. This therefore compensated for the effect of SHO in the subsequent developmental phases. Thus, we assume that these QTLs play a role in very early plant development by affecting the time to reach SHO and the duration of SEL.

We also observed QTLs specific to the SEL trait. For instance, QTL-2H-10 extended SEL by 19.7 GDD, while QTL-4H-5, QTL-5H-2 and QTL-5H-7 shortened SEL by 12.2, 12.1 and 18.0 GDD, respectively. Interestingly, some of the above-mentioned QTLs also affected TGW and HEI in addition to affecting SEL. In the case of QTL-5H-2, the shortened period of SEL correlates with reduced HEI. Although QTL-4H-5 and QTL-2H-10 show contrasting effects on SEL, they co-localized with QTLs that decrease TGW. In the case of QTL-2H-10 this effect is likely caused by the six-rowed spike locus *Vrs1* (Komatsuda *et al.*, 2007) originating from the *Hordeum vulgare* ssp. *agriocrithon* donor of HEB-F24 in our population. Six-rowed spikes generally show decreased TGW due to a higher grain number. This causes single grains to compete for assimilates. Interestingly, *Vrs1* also seems to extend SEL. This corroborates the observation that six-rowed barley has a higher leaf area and leaf dry weight than two-rowed barley, since leaf biomass is mainly produced during SEL (Alqudah and Schnurbusch, 2015).

### QTLs affecting late development

QTLs that affect late development are thought to influence RIP and MAT. Following Davies and Gan (2012), whole plant senescence is initiated through a physiological transition at flowering. This leads to a remobilization of nutrients to the developing seeds (Distelfeld *et al.*, 2014). In the present study, RIP represents the period of whole plant senescence and grain-filling. Thus, it is considered to be highly important in determining yield, particularly as a result of the grain weight component (Distelfeld *et al.*, 2014). Egli (2004) also emphasized that the duration of this period played an important role in grain crop yields.

The most obvious QTL to specifically affect late development was detected in the centromeric region of chromosome 6H (QTL-6H-4). In addition to affecting RIP and MAT, this QTL also significantly affected TGW and HEI. *HvNAM-1* (*NO APICAL MERISTEM-1*), coding for an NAC transcription factor (Distelfeld *et al.*, 2008), is a promising candidate for this locus. It plays an important role in inducing senescence (Distelfeld *et al.*, 2014). Moreover, it has been shown that its wheat homologue *NAM-B1* influences the remobilization process of nitrogen, iron and zinc in the developing grain (Uauy *et al.*, 2006; Waters *et al.*, 2009). In our study the wild allele accelerated senescence, which shortened RIP by approximately 1 day and consequently led to earlier MAT (–3.4 days). Simultaneously, TGW was reduced by 2.6g and HEI was reduced by 4.5cm. Interestingly, there is a second QTL for RIP (QTL-6H-5) that is closely linked to *HvNAM-1*. It mainly affects early stages by delaying SHO and HEA and shortening RIP. This behaviour points to the candidate gene *HvGR-RBP1* (*GLYCINE-RICH RNA BINDING PROTEIN*), which is consistent with the findings of other groups (Jukanti *et al.*, 2008; Streitner *et al.*, 2008; Mason *et al.*, 2014). This is the first time in barley genomics that we have been able to clearly distinguish this adjacent locus from *HvNAM-1*.

### Comparison of shared QTLs

In previous sections we mentioned many QTLs that simultaneously affected several developmental traits. Comparing their effects across traits provides insights into the different modes of action of the respective QTLs (Fig. 1 and Table 5).

In general, most QTLs affecting HEA also have a similar effect on SHO. This is also reflected by the high correlation of *r*=0.92 (Table 3). However, comparing the absolute effects on both stages, we can state that the effect on HEA is generally more pronounced. This is reflected by an increased SEL and indicates that the effect may accumulate over time. However, the *denso/sdw1* locus is a striking exception to this rule. Although both time to SHO and HEA are reduced, it simultaneously extends the time needed for SEL. This indicates that the effect on SHO is stronger than on HEA at *denso/sdw1*. We therefore assume that *denso/sdw1* has the biggest impact in the very early developmental stages and that its influence decreases during subsequent developmental phases.

Almost half of all developmental QTLs in our study simultaneously showed significant effects on HEI. However, the direction in which HEI was influenced differed between QTLs, even if they shared the same tendencies with regard to developmental traits. This is interesting when it comes to separating general developmental effects from purely reproduction-promoting effects. In most cases, HEI increased as the duration of SEL increased and vice versa. This is already indicated by the positive correlation of both traits (*r*=0.45). However, in the case of QTL-4H-1 and *Vrn-H2*, the prolonged duration of SEL is accompanied by a reduction in HEI. Since these QTLs retard development and simultaneously reduce HEI, we assume that their gene products may generally hamper plant development.

### Relevance of developmental subphases with regard to TGW

We observed numerous QTLs that were significantly associated with the developmental subphases SEL and RIP. We observed a general influence on TGW for the duration of grain-filling, i.e. RIP; as RIP increased, TGW increased as well. However, we could not find any direct correlations between SEL and TGW. There were hints that the interplay between RIP and SEL may be of interest. In general, we observed a negative correlation between both traits with *r*=–0.6. Consequently, a shortened SEL would cause an extended RIP. This would then cause a longer period of grain-filling and thus increase TGW. The time of physiological maturity (MAT) is largely predetermined by environmental factors, as discussed above. Therefore, increasing the duration of RIP leads to earlier flowering, and very likely also to a shorter period of SEL. Thus, if the duration of RIP is extended to increase TGW, this will result in a reduction in SEL. This lowers the amount of biomass that would be able to supply the developing grains with assimilates (Alqudah and Schnurbusch, 2015). Normally this reduction would not have serious consequences since plants produce excess vegetative mass (Egli, 1998). However, if the effect is severe (e.g. in the case of *Ppd-H1* in our study), the beneficial effect on TGW may be compensated. We therefore presume that increasing TGW is based on a trade-off between an extended RIP and a shortened SEL. However, one has to keep in mind that yield formation is based on a complex interplay between several yield components. To scrutinize our findings, we therefore suggest studying all yield components simultaneously, i.e. tiller number, number of grains per spike and grain weight, as well as total grain yield, in future HEB-25 field experiments. In addition, spike photosynthesis may also require analysis, since its role in grain-filling has recently been highlighted (Sanchez-Bragado *et al.*, 2014).

### Beneficial wild germplasm present in HEB-25

One major advantage of using HEB-25 is the ability to directly evaluate the value of wild barley alleles in a cultivated background. Thus, we were able to identify 10 QTLs where the wild allele increased TGW by up to 6.6g compared with the elite Barke allele. These wild QTL alleles are promising candidates for improving future barley grain weight once they are introduced into barley breeding programmes. However, the optimum strategy of adjusting plant developmental subphases to increase grain weight may vary depending on the ecogeographic region in which barley is grown. As latitudes increase, alleles conferring a decelerated plant development would be favourable for making use of the longer and cooler season for grain-filling. For barley cultivated in lower latitudes, early flowering would be favourable, for instance, to escape early season terminal drought (Comadran *et al.*, 2012). Therefore, it is currently not possible to make a general statement about the usefulness of specific plant development QTL effects. However, as we see that there is tremendous effect variation for every trait, we conclude that HEB-25 harbours a multitude of beneficial plant development QTL alleles for different ecogeographic regions.

In this study, the most prominent example for putative advantageous effects of wild alleles is the *denso/sdw1* QTL on chromosome 3H. Barke carries the dwarfing *denso* allele, which was introduced in barley breeding programmes during the ‘Green Revolution’. It reduces plant height and improves resistance to lodging, whereby also yield is increased (Jia *et al.*, 2009). The wild allele extends SEL and RIP, and increases TGW, compared with the cultivated Barke allele. Unfortunately, these beneficial effects are accompanied by the unfavourable effect of increasing HEI. However, we observed lines (e.g. HEB_25_050) in HEB-25 that carry the wild allele at the *denso/sdw1* locus and nevertheless demonstrate high agronomic performance. This is most likely due to introgression of additional wild alleles, which appear to compensate for the effect of increasing HEI (Supplementary Table S6). We thus encourage breeders to integrate wild germplasm into their breeding programmes. This way, the elite barley gene pool can be replenished with new favourable alleles to overcome future agricultural challenges.

## Supplementary data

Supplementary data are available at *JXB* online.

Figure S1. Frequency distributions of BLUEs for all investigated traits, plotted as histograms.

Figure S2. Venn diagrams indicating the number of shared QTLs across traits.

Table S1. Results of BARLEYMAP alignments to detect QTL candidate genes.

Table S2. Descriptive statistics of all investigated traits, grouped by years.

Table S3. Heritabilities (*h* ^2^) of all investigated traits, including the comparison of days and GDD for developmental traits.

Table S4. Tabular overview of all results gathered from GWAS, along with family-specific effect estimates for each SNP.

Table S5. Overview of all QTLs and their effects, with position, estimated effect on the different traits, and plausible candidate genes.

Table S6. Raw phenotype data and BLUEs of the investigated traits across years and blocks, along with information about the allelic state at the *denso/sdw1* locus.

Supplementary Data

## References

[CIT0001] Alonso-Peral MM Oliver SN Casao MC Greenup AA Trevaskis B 2011 The promoter of the cereal VERNALIZATION1 gene is sufficient for transcriptional induction by prolonged cold. PLoS ONE 6, e29456. 22242122 10.1371/journal.pone.0029456 PMC3248443

[CIT0002] Alqudah AM Schnurbusch T 2014 Awn primordium to tipping is the most decisive developmental phase for spikelet survival in barley. Functional Plant Biology 41, 424–436. 10.1071/FP13248 32481002

[CIT0003] Alqudah AM Schnurbusch T 2015 Barley leaf area and leaf growth rates are maximized during the pre-anthesis phase. Agronomy 5, 107–129.

[CIT0004] Alqudah AM Sharma R Pasam RK Graner A Kilian B Schnurbusch T 2014 Genetic dissection of photoperiod response based on GWAS of pre-anthesis phase duration in spring barley. PLoS ONE 9, e113120. 25420105 10.1371/journal.pone.0113120 PMC4242610

[CIT0005] Atkin OK Tjoelker MG 2003 Thermal acclimation and the dynamic response of plant respiration to temperature. Trends in Plant Science 8, 343–351. 12878019 10.1016/S1360-1385(03)00136-5

[CIT0006] Atwell BJ Kriedemann PE Turnbull CGN 1999 *Plants in action: adaptation in nature, performance in cultivation*. South Yarra, Victoria: Macmillan Education Australia.

[CIT0007] Blümel M Dally N Jung C 2015 Flowering time regulation in crops—what did we learn from Arabidopsis? Current Opinion in Biotechnology 32, 121–129. 25553537 10.1016/j.copbio.2014.11.023

[CIT0008] Boden SA Weiss D Ross JJ Davies NW Trevaskis B Chandler PM Swain SM 2014 EARLY FLOWERING3 regulates flowering in spring barley by mediating gibberellin production and FLOWERING LOCUS T expression. *The Plant Cell* 26, 1557–1569. 10.1105/tpc.114.123794 PMC4036571 24781117

[CIT0009] Borras-Gelonch G Denti M Thomas WTB Romagosa I 2012 Genetic control of pre-heading phases in the Steptoe x Morex barley population under different conditions of photoperiod and temperature. Euphytica 183, 303–321.

[CIT0010] Borras-Gelonch G Slafer GA Casas AM van Eeuwijk F Romagosa I 2010 Genetic control of pre-heading phases and other traits related to development in a double-haploid barley (Hordeum vulgare L.) population. Field Crops Research 119, 36–47.

[CIT0011] Borras G Romagosa I van Eeuwijk F Slafer GA 2009 Genetic variability in duration of pre-heading phases and relationships with leaf appearance and tillering dynamics in a barley population. Field Crops Research 113, 95–104.

[CIT0012] Buckler ES Holland JB Bradbury PJ 2009 The genetic architecture of maize flowering time. Science 325, 714–718. 19661422 10.1126/science.1174276

[CIT0013] Calixto CPG Waugh R Brown JWS 2015 Evolutionary relationships among barley and Arabidopsis core circadian clock and clock-associated genes. Journal of Molecular Evolution 80, 108–119. 25608480 10.1007/s00239-015-9665-0 PMC4320304

[CIT0014] Campoli C Pankin A Drosse B Casao CM Davis SJ Korff M 2013 HvLUX1 is a candidate gene underlying the early maturity 10 locus in barley: phylogeny, diversity, and interactions with the circadian clock and photoperiodic pathways. New Phytologist 199, 1045–1059. 23731278 10.1111/nph.12346 PMC3902989

[CIT0015] Campoli C Shtaya M Davis SJ von Korff M 2012 Expression conservation within the circadian clock of a monocot: natural variation at barley Ppd-H1 affects circadian expression of flowering time genes, but not clock orthologs. BMC Plant Biology 12, 97. 22720803 10.1186/1471-2229-12-97 PMC3478166

[CIT0016] Cantalapiedra CP Boudiar R Casas AM Igartua E Contreras-Moreira B 2015 BARLEYMAP: physical and genetic mapping of nucleotide sequences and annotation of surrounding loci in barley. Molecular Breeding 35, 1–11.

[CIT0017] Chailakhyan MK 1937 *Gormonal’naya teoriya razvitiya rastenii* (Hormonal theory of plant development) . Moscow: Akademii Nauk SSSR.

[CIT0018] Cockram J Jones H O’Sullivan DM 2011 Genetic variation at flowering time loci in wild and cultivated barley. Plant Genetic Resources: Characterization and Utilization 9, 264–267.

[CIT0019] Cockram J Thiel T Steuernagel B Stein N Taudien S Bailey PC O’Sullivan DM 2012 Genome dynamics explain the evolution of flowering time CCT domain gene families in the Poaceae. PLoS ONE 7, e45307. 23028921 10.1371/journal.pone.0045307 PMC3454399

[CIT0020] Comadran J Kilian B Russell J 2012 Natural variation in a homolog of Antirrhinum CENTRORADIALIS contributed to spring growth habit and environmental adaptation in cultivated barley. Nature Genetics 44, 1388–1392. 23160098 10.1038/ng.2447

[CIT0021] Davies P Gan S 2012 Towards an integrated view of monocarpic plant senescence. Russian Journal of Plant Physiology 59, 467–478.

[CIT0022] Deng W Casao MC Wang P Sato K Hayes PM Finnegan EJ Trevaskis B 2015 Direct links between the vernalization response and other key traits of cereal crops. Nature Communications 6, 5882. 10.1038/ncomms6882 25562483

[CIT0023] Distelfeld A Avni R Fischer AM 2014 Senescence, nutrient remobilization, and yield in wheat and barley. Journal of Experimental Botany 65, 3783–3798. 24470467 10.1093/jxb/ert477

[CIT0024] Distelfeld A Korol A Dubcovsky J Uauy C Blake T Fahima T 2008 Colinearity between the barley grain protein content (GPC) QTL on chromosome arm 6HS and the wheat Gpc-B1 region. Molecular Breeding 22, 25–38.

[CIT0025] Draper N Smith H 1981 *Applied regression analysis* . New York: John Wiley.

[CIT0026] Egli DB 1998 *Seed biology and the yield of grain crops* . Wallingford, Oxon: CAB International.

[CIT0027] Egli DB 2004 Seed-fill duration and yield of grain crops. Advances in Agronomy 83, 243–279.

[CIT0028] Evans LT Wardlaw IF 1976 Aspects of the comparative physilogy of grain yield in cereals. Advances in Agronomy 28, 301–359.

[CIT0029] Faure S Higgins J Turner A Laurie DA 2007 The FLOWERING LOCUS T-like gene family in barley (Hordeum vulgare). Genetics 176, 599–609. 17339225 10.1534/genetics.106.069500 PMC1893030

[CIT0030] Faure S Turner AS Gruszka D Christodoulou V Davis SJ von Korff M Laurie DA 2012 Mutation at the circadian clock gene EARLY MATURITY 8 adapts domesticated barley (Hordeum vulgare) to short growing seasons. Proceedings of the National Academy of Sciences of the United States of America 109, 8328–8333. 22566625 10.1073/pnas.1120496109 PMC3361427

[CIT0031] Franckowiak J Lundqvist U Konishi T Gallagher L 1997 Description of stock number: BGS 214. Barley Genetics Newsletter 26, 213–215.

[CIT0032] Holm S 1979 A simple sequentially rejective multiple test procedure. Scandinavian Journal of Statistics 6, 65–70.

[CIT0033] International Barley Genome Sequencing Consortium 2012 A physical, genetic and functional sequence assembly of the barley genome. Nature 491, 711–716. 23075845 10.1038/nature11543

[CIT0034] Jia Q Zhang X-Q Westcott S Broughton S Cakir M Yang J Lance R Li C 2011 Expression level of a gibberellin 20-oxidase gene is associated with multiple agronomic and quality traits in barley. Theoretical and Applied Genetics 122, 1451–1460. 21318371 10.1007/s00122-011-1544-5

[CIT0035] Jia Q Zhang J Westcott S Zhang X-Q Bellgard M Lance R Li C 2009 GA-20 oxidase as a candidate for the semidwarf gene sdw1/denso in barley. Functional & Integrative Genomics 9, 255–262. 19280236 10.1007/s10142-009-0120-4

[CIT0036] Jordan D Mace E Cruickshank A Hunt C Henzell R 2011 Exploring and exploiting genetic variation from unadapted sorghum germplasm in a breeding program. Crop Science 51, 1444–1457.

[CIT0037] Jukanti AK Heidlebaugh NM Parrott DL Fischer IA McInnerney K Fischer AM 2008 Comparative transcriptome profiling of near‐isogenic barley (Hordeum vulgare) lines differing in the allelic state of a major grain protein content locus identifies genes with possible roles in leaf senescence and nitrogen reallocation. New Phytologist 177, 333–349. 18028296 10.1111/j.1469-8137.2007.02270.x

[CIT0038] Kernich GC Halloran GM Flood RG 1997 Variation in duration of pre-anthesis phases of development in barley (Hordeum vulgare). Australian Journal of Agricultural Research 48, 59–66.

[CIT0039] Kikuchi R Kawahigashi H Ando T Tonooka T Handa H 2009 Molecular and functional characterization of PEBP genes in barley reveal the diversification of their roles in flowering. Plant Physiology 149, 1341–1353. 19168644 10.1104/pp.108.132134 PMC2649388

[CIT0040] Komatsuda T Pourkheirandish M He C Azhaguvel P Kanamori H Perovic D Stein N Graner A Wicker T Tagiri A 2007 Six-rowed barley originated from a mutation in a homeodomain-leucine zipper I-class homeobox gene. Proceedings of the National Academy of Sciences of the United States of America 104, 1424–1429. 17220272 10.1073/pnas.0608580104 PMC1783110

[CIT0041] Kurakawa T Ueda N Maekawa M Kobayashi K Kojima M Nagato Y Sakakibara H Kyozuka J 2007 Direct control of shoot meristem activity by a cytokinin-activating enzyme. Nature 445, 652–655. 17287810 10.1038/nature05504

[CIT0042] Lancashire PD Bleiholder H Boom TVD Langelüddeke P Stauss R Weber E Witzenberger A 1991 A uniform decimal code for growth stages of crops and weeds. Annals of Applied Biology 119, 561–601.

[CIT0043] Laurie D Pratchett N Snape J Bezant J 1995 RFLP mapping of five major genes and eight quantitative trait loci controlling flowering time in a winter × spring barley (Hordeum vulgare L.) cross. Genome 38, 575–585. 18470191 10.1139/g95-074

[CIT0044] Liu W Gowda M Steinhoff J Maurer HP Würschum T Longin CFH Cossic F Reif JC 2011 Association mapping in an elite maize breeding population. Theoretical and Applied Genetics 123, 847–858. 21681489 10.1007/s00122-011-1631-7

[CIT0045] Loscos J Igartua E Contreras-Moreira B Gracia MP Casas AM 2014 HvFT1 polymorphism and effect – survey of barley germplasm and expression analysis. Frontiers in Plant Science 5, 251. 24936204 10.3389/fpls.2014.00251 PMC4047512

[CIT0046] Mascher M Muehlbauer GJ Rokhsar DS 2013 Anchoring and ordering NGS contig assemblies by population sequencing (POPSEQ). The Plant Journal 76, 718–727. 23998490 10.1111/tpj.12319 PMC4298792

[CIT0047] Mason KE Tripet BP Parrott D Fischer AM Copié V 2014 1H, 13C, 15N backbone and side chain NMR resonance assignments for the N-terminal RNA recognition motif of the HvGR-RBP1 protein involved in the regulation of barley (Hordeum vulgare L.) senescence. Biomolecular NMR Assignments 8, 149–153. 23417794 10.1007/s12104-013-9472-8 PMC3672310

[CIT0048] Maurer A Draba V Jiang Y Schnaithmann F Sharma R Schumann E Kilian B Reif JC Pillen K 2015 Modelling the genetic architecture of flowering time control in barley through nested association mapping. BMC Genomics 16, 290. 25887319 10.1186/s12864-015-1459-7 PMC4426605

[CIT0049] McMaster GS Smika DE 1988 Estimation and evaluation of winter wheat phenology in the central Great Plains. Agricultural and Forest Meteorology 43, 1–18.

[CIT0050] McMaster GS Wilhelm W 1997 Growing degree-days: one equation, two interpretations. Agricultural and Forest Meteorology 87, 291–300.

[CIT0051] Miralles DJ Richards RA Slafer GA 2000 Duration of the stem elongation period influences the number of fertile florets in wheat and barley. Functional Plant Biology 27, 931–940.

[CIT0052] Mrízová K Jiskrová E Vyroubalová Š Novák O Ohnoutková L Pospíšilová H Frébort I Harwood WA Galuszka P 2013 Overexpression of cytokinin dehydrogenase genes in barley (Hordeum vulgare cv. Golden Promise) fundamentally affects morphology and fertility. PLoS ONE 8, e79029. 24260147 10.1371/journal.pone.0079029 PMC3829838

[CIT0053] Mutasa-Göttgens E Hedden P 2009 Gibberellin as a factor in floral regulatory networks. Journal of Experimental Botany 60, 1979–1989. 19264752 10.1093/jxb/erp040

[CIT0054] Nishida H Ishihara D Ishii M Kaneko T Kawahigashi H Akashi Y Saisho D Tanaka K Handa H Takeda K 2013 Phytochrome C is a key factor controlling long-day flowering in barley. Plant Physiology 163, 804–814. 24014575 10.1104/pp.113.222570 PMC3793059

[CIT0055] Ogut F Bian Y Bradbury P Holland J 2015 Joint-multiple family linkage analysis predicts within-family variation better than single-family analysis of the maize nested association mapping population. Heredity 114, 1–12. 25585918 10.1038/hdy.2014.123 PMC4434247

[CIT0056] Oliver SN Deng W Casao MC Trevaskis B 2013 Low temperatures induce rapid changes in chromatin state and transcript levels of the cereal VERNALIZATION1 gene. Journal of Experimental Botany 64, 2413–2422. 23580755 10.1093/jxb/ert095 PMC3654426

[CIT0057] Pankin A Campoli C Dong X Kilian B Sharma R Himmelbach A Saini R Davis SJ Stein N Schneeberger K 2014 Mapping-by-sequencing identifies HvPHYTOCHROME C as a candidate gene for the early maturity 5 locus modulating the circadian clock and photoperiodic flowering in barley. Genetics 198, 383–396. 24996910 10.1534/genetics.114.165613 PMC4174949

[CIT0058] Sanchez-Bragado R Molero G Reynolds MP Araus JL 2014 Relative contribution of shoot and ear photosynthesis to grain filling in wheat under good agronomical conditions assessed by differential organ δ13C. Journal of Experimental Botany 65, 5401–5413. 25053645 10.1093/jxb/eru298 PMC4157716

[CIT0059] Schwarz G 1978 Estimating the dimension of a model. The Annals of Statistics 6, 461–464.

[CIT0060] Sreenivasulu N Schnurbusch T 2012 A genetic playground for enhancing grain number in cereals. Trends in Plant Science 17, 91–101. 22197176 10.1016/j.tplants.2011.11.003

[CIT0061] Streitner C Danisman S Wehrle F Schöning JC Alfano JR Staiger D 2008 The small glycine-rich RNA binding protein AtGRP7 promotes floral transition in Arabidopsis thaliana. The Plant Journal 56, 239–250. 18573194 10.1111/j.1365-313X.2008.03591.x

[CIT0062] Taiz L Zeiger E 2007 *Plant physiology* . Heidelberg: Spektrum Akademischer Verlag/Springer.

[CIT0063] Turck F Fornara F Coupland G 2008 Regulation and identity of florigen: FLOWERING LOCUS T moves center stage. Annual Review of Plant Biology 59, 573–594. 10.1146/annurev.arplant.59.032607.092755 18444908

[CIT0064] Turner A Beales J Faure S Dunford RP Laurie DA 2005 The pseudo-response regulator Ppd-H1 provides adaptation to photoperiod in barley. Science 310, 1031–1034. 16284181 10.1126/science.1117619

[CIT0065] Uauy C Distelfeld A Fahima T Blechl A Dubcovsky J 2006 A NAC gene regulating senescence improves grain protein, zinc, and iron content in wheat. Science 314, 1298–1301. 17124321 10.1126/science.1133649 PMC4737439

[CIT0066] von Zitzewitz J Szűcs P Dubcovsky J Yan L Francia E Pecchioni N Casas A Chen TH Hayes PM Skinner JS 2005 Molecular and structural characterization of barley vernalization genes. Plant Molecular Biology 59, 449–467. 16235110 10.1007/s11103-005-0351-2

[CIT0067] Wang D-Y Li Q Cui K-M Zhu Y-X 2007 Gibberellin is involved in the regulation of cell death‐mediated apical senescence in G2 pea. Journal of Integrative Plant Biology 49, 1627–1633.

[CIT0068] Wang G Schmalenbach I von Korff M Leon J Kilian B Rode J Pillen K 2010 Association of barley photoperiod and vernalization genes with QTLs for flowering time and agronomic traits in a BC(2)DH population and a set of wild barley introgression lines. Theoretical and Applied Genetics 120, 1559–1574. 20155245 10.1007/s00122-010-1276-y PMC2859222

[CIT0069] Waters BM Uauy C Dubcovsky J Grusak MA 2009 Wheat (Triticum aestivum) NAM proteins regulate the translocation of iron, zinc, and nitrogen compounds from vegetative tissues to grain. Journal of Experimental Botany 60, 4263–4274. 19858116 10.1093/jxb/erp257

[CIT0070] Weier D Thiel J Kohl S Tarkowská D Strnad M Schaarschmidt S Weschke W Weber H Hause B 2014 Gibberellin-to-abscisic acid balances govern development and differentiation of the nucellar projection of barley grains. Journal of Experimental Botany 65, 5291–5304. 25024168 10.1093/jxb/eru289 PMC4157710

[CIT0071] Würschum T Liu W Gowda M Maurer H Fischer S Schechert A Reif J 2012 Comparison of biometrical models for joint linkage association mapping. Heredity 108, 332–340. 21878984 10.1038/hdy.2011.78 PMC3282402

[CIT0072] Yan L Fu D Li C Blechl A Tranquilli G Bonafede M Sanchez A Valarik M Yasuda S Dubcovsky J 2006 The wheat and barley vernalization gene VRN3 is an orthologue of FT. Proceedings of the National Academy of Sciences of the United States of America 103, 19581–19586. 17158798 10.1073/pnas.0607142103 PMC1748268

[CIT0073] Yan L Loukoianov A Blechl A Tranquilli G Ramakrishna W SanMiguel P Bennetzen JL Echenique V Dubcovsky J 2004 The wheat VRN2 gene is a flowering repressor down-regulated by vernalization. Science 303, 1640–1644. 15016992 10.1126/science.1094305 PMC4737501

[CIT0074] Yan L Loukoianov A Tranquilli G Helguera M Fahima T Dubcovsky J 2003 Positional cloning of the wheat vernalization gene VRN1. Proceedings of the National Academy of Sciences of the United States of America 100, 6263–6268. 12730378 10.1073/pnas.0937399100 PMC156360

[CIT0075] Zakhrabekova S Gough SP Braumann I 2012 Induced mutations in circadian clock regulator Mat-a facilitated short-season adaptation and range extension in cultivated barley. Proceedings of the National Academy of Sciences of the United States of America 109, 4326–4331. 22371569 10.1073/pnas.1113009109 PMC3306670

