# Supplementary material for: Genomic dissection of plant development and its impact on thousand grain weight in barley through nested association mapping
Source: J Exp Bot. 2016 Mar 1;67(8):2507–18. doi: 10.1093/jxb/erw070 (PMC4809299; doi:10.1093/jxb/erw070)
Supplement: Supplementary Data [file supp_67_8_2507__index.html]

Genomic dissection of plant development and its impact on thousand grain weight in barley through nested association mapping — Genomic dissection of plant development and its impact on thousand grain weight in barley through nested association mapping — Supplementary Data 

# Genomic dissection of plant development and its impact on thousand grain weight in barley through nested association mapping

## Supplementary Data

Data files

- supplementary\_figures\_S1\_S2.pdf - Supplementary Data
- supplementary\_tables\_S1\_S6.xlsx - Supplementary Data
