# Supplementary material for: Genomic dissection of plant development and its impact on thousand grain weight in barley through nested association mapping
Source: J Exp Bot. 2016 Mar 1;67(8):2507–18. doi: 10.1093/jxb/erw070 (PMC4809299; doi:10.1093/jxb/erw070)
Supplement: Supplementary Data [file supp_erw070_supplementary_figures_S1_S2.pdf]

Fig. S1) Frequency distributions of BLUEs for all investigated traits

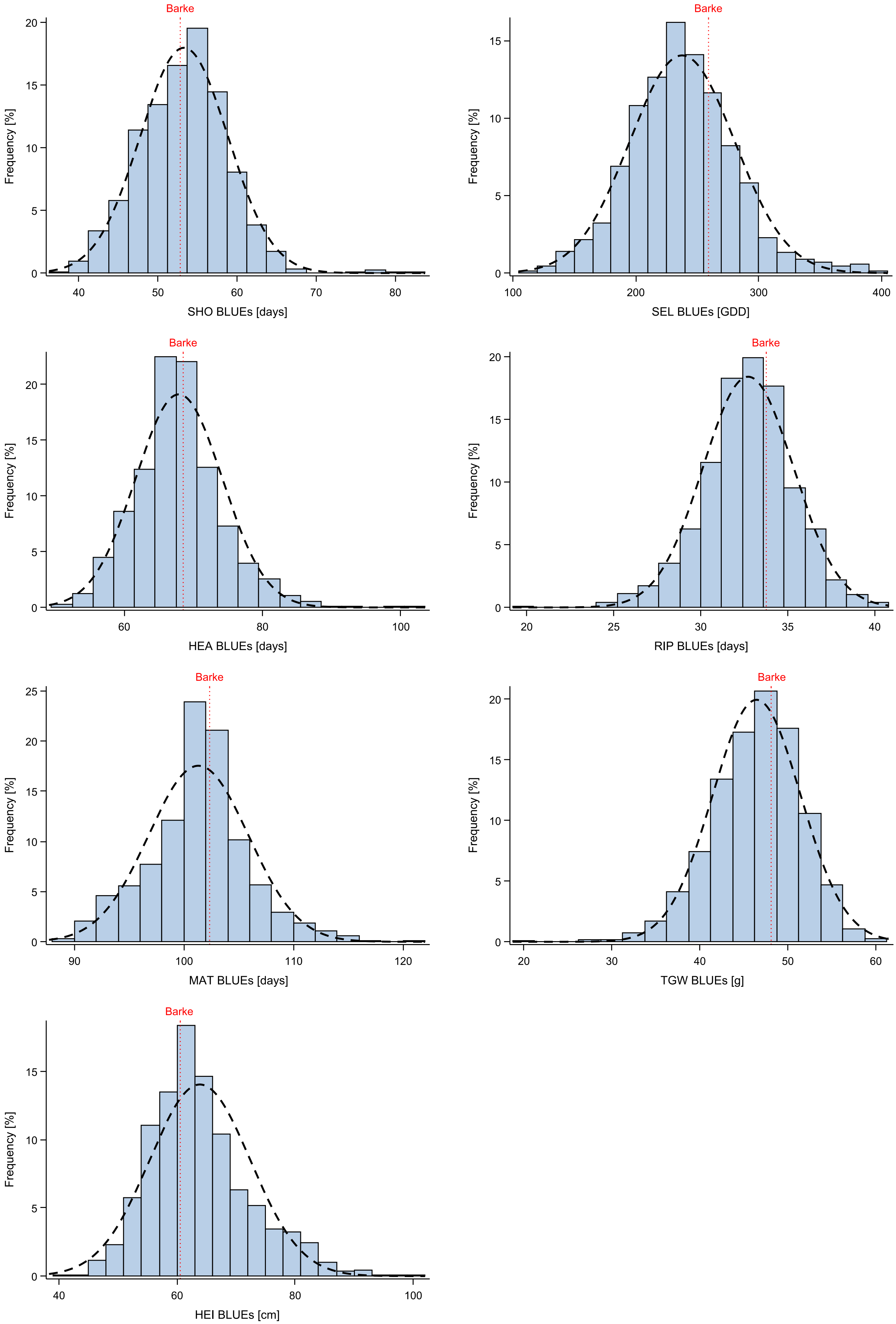

Figure S2) Venn diagrams of shared QTL across traits.

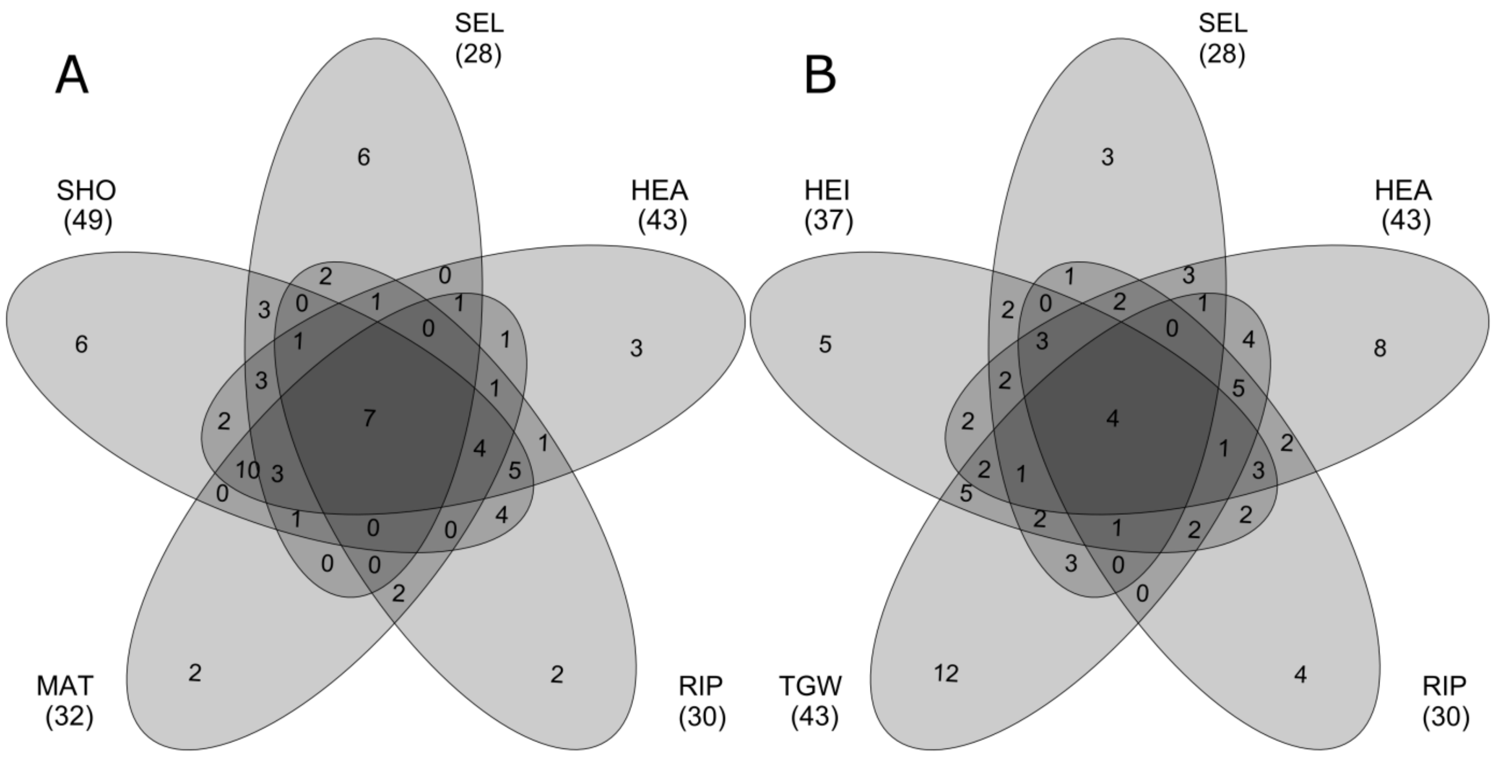

Different traits are represented by elliptic areas and numbers in their overlap indicate the number of shared QTL. Numbers in the outer non-overlapping regions represent trait-specific QTL. Numbers in brackets indicate the total number of QTL per trait. **A)** Shared QTL across the developmental traits SHO, SEL, HEA, RIP and MAT. **B)** Shared QTL across the developmental traits SEL, HEA and RIP, as well as TGW and HEI.
